# Dysfunctional high-density lipoprotein activates toll-like receptors via serum amyloid A in vascular smooth muscle cells

**DOI:** 10.1038/s41598-019-39846-3

**Published:** 2019-03-04

**Authors:** Mirjam Schuchardt, Nicole Prüfer, Yuexing Tu, Jaqueline Herrmann, Xiu-Ping Hu, Sarah Chebli, Katja Dahlke, Walter Zidek, Markus van der Giet, Markus Tölle

**Affiliations:** 1 Charité – Universitätsmedizin Berlin, corporate member of Freie Universität Berlin, Humboldt Universität zu Berlin, and Berlin Institute of Health, Department of Nephrology, Hindenburgdamm 30, 12203 Berlin, Germany; 2 Zhejiang Provincial People´s Hospital, Intensive Care Unit, Hangzhou, China; 3 Deutsches Institut für Ernaehrungsforschung, Department of Gastrointestinal Microbiology, Arthur-Scheunert-Allee 114-116, 14558 Nuthethal, Germany

## Abstract

Serum amyloid A (SAA) is an uremic toxin and acute phase protein. It accumulates under inflammatory conditions associated with high cardiovascular morbidity and mortality in patients with sepsis or end-stage renal disease (ESRD). SAA is an apolipoprotein of the high-density lipoprotein (HDL). SAA accumulation turns HDL from an anti-inflammatory to a pro-inflammatory particle. SAA activates monocyte chemoattractant protein-1 (MCP-1) in vascular smooth muscle cells. However, the SAA receptor-mediated signaling pathway in vascular cells is poorly understood. Therefore, the SAA-mediated signaling pathway for MCP-1 production was investigated in this study. The SAA-induced MCP-1 production is dependent on the activation of TLR2 and TLR4 as determined by studies with specific receptor antagonists and agonists or siRNA approach. Experiments were confirmed in tissues from TLR2 knockout, TLR4 deficient and TLR2 knock-out/TLR4 deficient mice. The intracellular signaling pathway is IκBα and subsequently NFκB dependent. The MCP-1 production induced by SAA-enriched HDL and HDL isolated from septic patients with high SAA content is also TLR2 and TLR4 dependent. Taken together, the TLR2 and TLR4 receptors are functional SAA receptors mediating MCP-1 release. Furthermore, the TLR2 and TLR4 are receptors for dysfunctional HDL. These results give a further inside in SAA as uremic toxin involved in uremia-related pro-inflammatory response in the vascular wall.

## Introduction

Serum amyloid A (SAA) is a highly conserved acute-phase protein predominantly synthesized by the liver^1^. The precise physiological and pathophysiological role of SAA in acute and chronic inflammatory disorders is not known so far. It is well known that SAA plasma levels are elevated during septic conditions^2^. Other findings support the role of SAA in cardiovascular diseases and atherosclerosis^3–5^. SAA is involved in immune defense mechanisms via acting chemotactic to different cell types and binding to gram-negative bacteria^1^. Furthermore, SAA plasma levels are significantly increased in patients with chronic kidney disease (CKD) and end-stage renal disease (ESRD)^6–8^ where an independent correlation with the cardiovascular risk exists^8^. Cardiovascular disease is the leading cause of death in patients with CKD. The mortality rate is up to 30-fold increased in patients with ESRD compared to renal-healthy patients^9,10^. Once released into the blood circulation, the apolipoprotein SAA mainly incorporates into high-density lipoprotein (HDL), mainly HDL_3_, but it can also be identified in low amounts in low-density lipoprotein (LDL) and very low-density lipoprotein (VLDL)^1,11^. As a consequence, SAA-loaded HDL changes its composition during uremic condition resulting in a less anti-inflammatory capacity^12^. SAA incorporation is associated with disturbed metabolism^13,14^ and decreased anti-inflammatory capacity of HDL especially from uremic patients^6,7^.

During inflammation, SAA expression and secretion is induced by several mediators in different cells, like macrophages, endothelial cells, and vascular smooth muscle cells (VSMC)^1^. Four different isoforms of SAA are found in the genome: SAA1, SAA2, SAA3, and SAA4^1^. In humans, SAA3 is a pseudo gene^1^. While SAA4 is constitutively produced, SAA1 and SAA2 serum concentration increases up to 1000-fold under acute inflammatory conditions with an approximate half-life of 24 h^1^.

As *in vitro* studies using recombinant human SAA (rhSAA) suggest, several structural diverse cell surface receptors are activated by SAA^1^. Up to now, at least 7 receptors have been identified: formyl-peptide receptor-like 1 (FPR2)^6,15,16^, toll-like receptor 2 and 4 (TLR2, TLR4)^17–20^, scavenger receptor type B-I (SR-BI)^21^, CD36^22,23^, receptor for advanced glycation end-products (RAGE)^20,24,25^, and the purinoceptor P2X_7_^26^.

The aim of this study was to examine the role of these receptors, especially TLR2 and TLR4, for chemokine activation in VSMC and macrophages. Our results indicate that beside FPR2 activation^6^ the TLR2 and TLR4 regulate the stimulation of MCP-1 production.

## Results

### HDL-bound-SAA induces MCP-1 production

**Figure 1:**
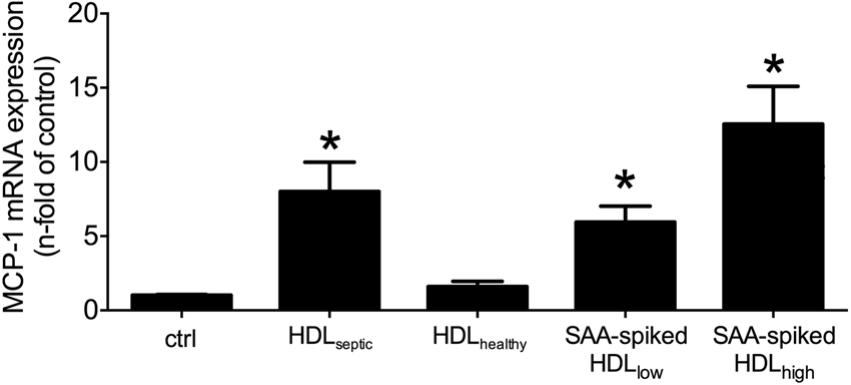
HDL from septic patients and SAA-spiked HDL from healthy controls induce MCP-1 mRNA expression. MCP-1 mRNA expression in rVSMC upon HDL (100 µg/ml) stimulation for 4 h. Data are presented as means ± SEM. *p < 0.05 compared to control (ctrl.).

From previous studies it was known that SAA enrichment of HDL in CKD patients turns this lipid particle to a pro-inflammatory state^6,7^. Stimulation of rVSMC with HDL from septic patients containing a high SAA concentration significantly induced MCP-1 mRNA expression compared to HDL from healthy subjects (Fig. 1). When HDL from healthy subjects was spiked with recombinant SAA in different dose, this artificial HDL-SAA also stimulated MCP-1 mRNA expression (Fig. 1).

The signaling pathway that is responsible for the SAA-induced pro-inflammatory reaction in VSMC is not entirely clear up to now. As known from a previous study, FPR2 is involved in SAA-mediated MCP-1 production in rVSMC^6^. However, the influence of other receptors activated by SAA remains open. Therefore, these receptors were investigated in the next step of this study.

### Receptor expression

**Figure 2:**
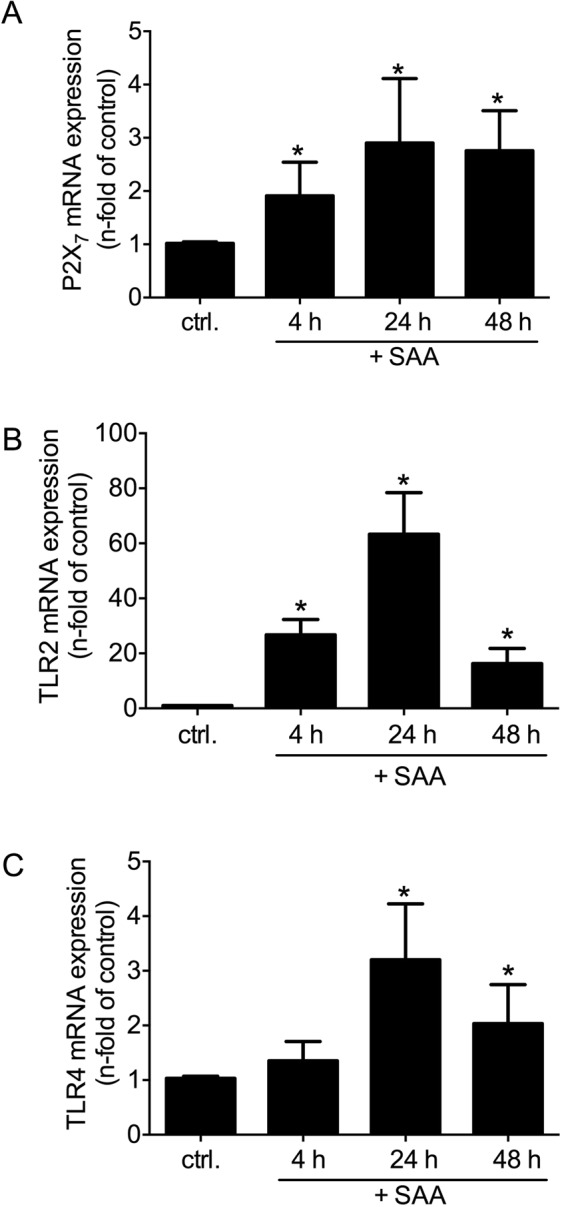
SAA-induced receptor expression. Time-dependent (**A**) P2X_7_, (**B**) TLR2 and (**C**) TLR4 receptor mRNA expression in rVSMC upon rhSAA (1 µg/ml) for 4, 24 and 48 h. Data are presented as means ± SEM. *p < 0.05 compared to control (ctrl.).

All of the known receptors (FPR2, TLR2, TLR4, SR-BI, CD36, RAGE, P2X_7_) potentially activated by SAA are expressed in rVSMC (Suppl. Fig. 1). First, the impact of SAA stimulation on receptor expression in rVSMC was investigated. After stimulation for 4 h, 24 h or 48 h, the mRNA expression levels of P2X_7_, TLR2, and TLR4 significantly increased compared to control (Fig. 2). The expression increase in TLR2 was the most abundant one and reached up to 67-fold after 24 h of stimulation. In contrast, the mRNA expression of CD36, SR-BI, or RAGE were not affected (Suppl. Fig. 2A–C). The expression of FPR2 was shown previously^6^.

### Influence of P2X_7_, TLR2 and TLR4 on SAA-mediated MCP-1 production in rVSMC

Given the involvement of P2X_7_, TLR2, and TLR4 in inflammatory conditions^27,28^ as well as its regulated expression upon SAA stimulation, we further characterized their role in MCP-1-mediated signaling.

**Figure 3:**
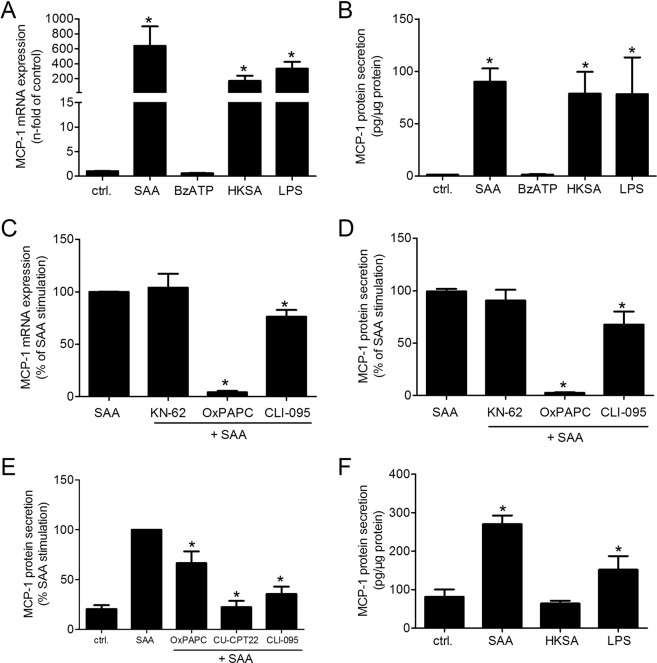
MCP-1 mRNA expression and protein secretion in rVSMC and THP-1 macrophage-like cell upon receptor agonists and antagonists. (**A**,**C**) MCP-1 mRNA expression and (**B**,**D**–**F**) MCP-1 protein secretion in (**A**–**D**) rVMSC or (**E**,**F**) THP-1 macrophage-like cells. RhSAA (1 µg/ml), BzATP (100 µmol/l), HKSA (10^7^ cells/ml), LPS (1 µg/ml), KN-62 (10 µmol/l), OxPAPC (30 µg/ml), CU-CPT22 (10 µmol/l), CLI-095 (10 µmol/l). Data are presented as means ± SEM. (**A,B,E,F**) *p < 0.05 compared to control (ctrl.) or (**C,D**) compared to SAA-stimulation.

To investigate viability effects of agonists and antagonists on cells, a cell viability assay based on metabolism was performed and showed no significant changes in the tested cells (Suppl. Fig. 3). The TLR2 agonist HKSA and the TLR4 agonist LPS significantly increased the MCP-1 mRNA expression and protein secretion in rVSMC, whereas the P2X_7_ agonist BzATP showed no significant effect (Fig. 3A,B). In line, the P2X_7_ antagonist KN-62 did not influence SAA-induced MCP-1 mRNA expression and protein secretion, whereas co-stimulation of rVSMC with the TLR4 antagonist CLI-095 and with the combined TLR2/4 antagonist OxPAPC significantly diminished MCP-1 mRNA expression and protein secretion (Fig. 3C,D). In line, antagonists for SR-BI (FPS-ZM1) or RAGE (BLT-1) had no influence on SAA-induced MCP-1 mRNA expression (Suppl. Fig. 2D,E). To confirm the effects of SAA on TLR2 and TLR4, a second cell line was used. In the macrophage like cell THP-1, the impact of TLR2 and TLR4 for MCP1 production could be verified. A significant MCP-1 protein secretion is induced by the TLR4 agonist LPS, but not by the TLR2 agonist HKSA. OxPAPC, CU-CPT22 and CLI-095 significantly diminished the SAA- and LPS-induced MCP-1 protein secretion in THP-1 cells (Fig. 3E,F).

### MCP-1 production in TLR2 and TLR4 knock-down cells and knock-out mice

**Figure 4:**
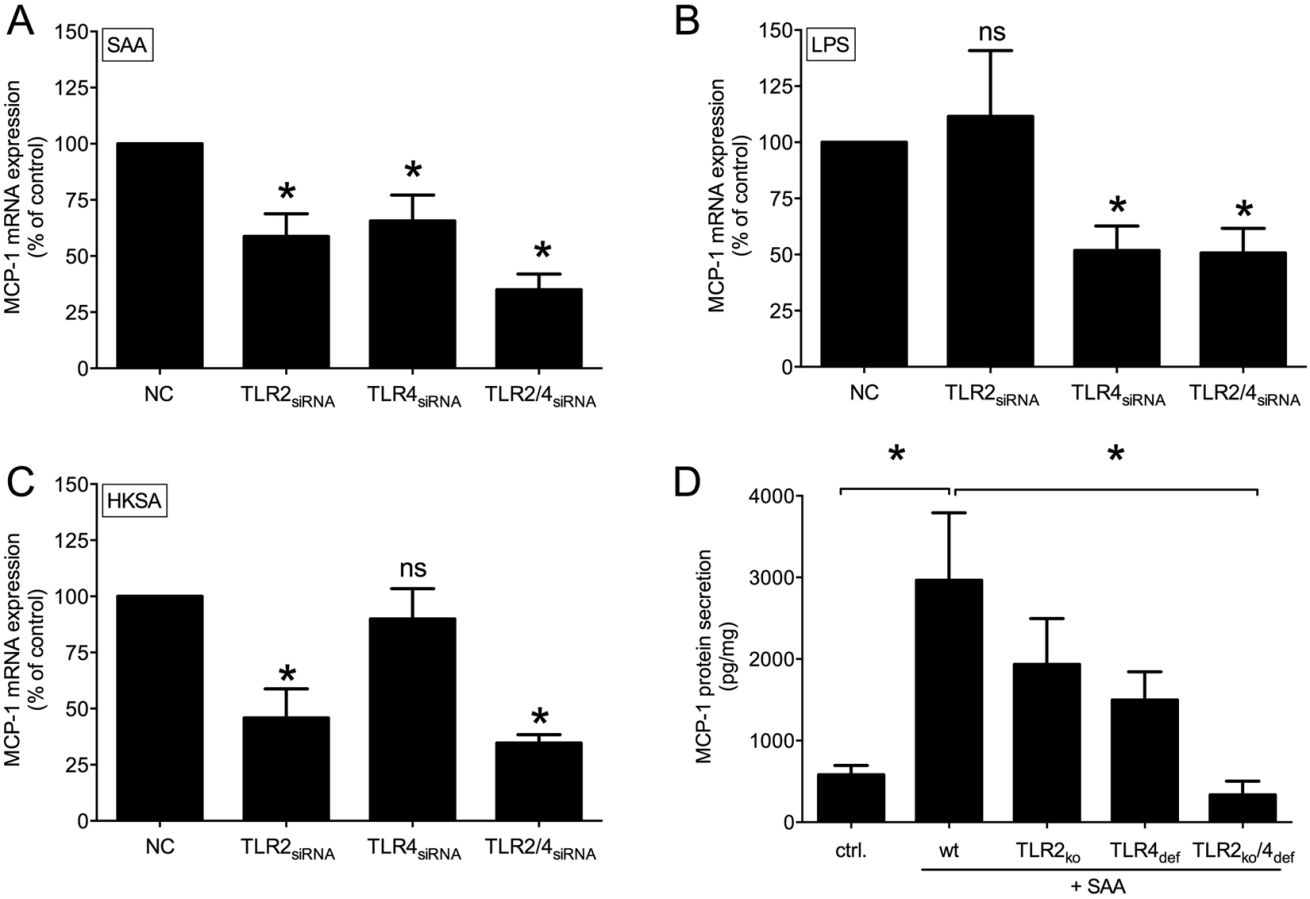
Effect of TLR2 and TLR4 verified by siRNA and receptor knockout model. (**A**–**C**) rVSMC were transfected with siRNA for TLR2 (TLR2_siRNA_), TLR4 (TLR4_siRNA_) or both (TLR2/4_siRNA_) as well as negative control siRNA (NC). Mock transfected cells were used as control in all experiments. Afterwards, cells were stimulated for 24 h with rhSAA (1 µg/ml), LPS (1 µg/ml) and HKSA (10^7^ cells/ml) with subsequent determination of MCP-1 mRNA expression. Data are presented as means ± SEM. *p < 0.05 compared to respective control. (**D**) Aortic tissue of TLR2 knockout (TLR2_ko_), TLR4 deficient (TLR4_def_) and TLR2_ko_/TLR4_def_ mice in comparison to control wild type littermates (wt) were stimulated *ex vivo* with rhSAA (1 µg/ml) for 24 h, respectively. MCP-1 protein secretion was determined in the supernatant and normalized to the aortic dry weight. Data are presented as means ± SEM. *p < 0.05 compared to unstimulated control (ctrl.), #p < 0.05 compared to wildtype littermate.

In order to confirm the role of TLR2 and TLR4 in SAA-mediated MCP-1 signaling in VSMCs, both receptors were silenced by siRNA approach. SAA-induced MCP-1 mRNA expression was inhibited in TLR2 and TLR4 single-knock-down cells (TLR2_siRNA_, TLR4_siRNA_) and double-knock-down cells (TLR2/4_siRNA_) after 24 hours of stimulation (Fig. 4A). LPS as TLR4 agonist induced MCP-1 mRNA expression in TLR2_siRNA_, but the effect was significantly diminished in TLR4_siRNA_ and TLR2/4_siRNA_ (Fig. 4B). In line, the TLR2 agonist HKSA induced MCP-1 mRNA expression in TLR4_siRNA_ but the effect was significantly reduced in TLR2_siRNA_ and TLR2/4_siRNA_ (Fig. 4C). These findings were confirmed in TLR2 knock-out (TLR2_ko_), TLR4 deficient (TLR4_def_) and combined TLR2_ko_/TLR4_def_ mice. While SAA stimulation of aortic rings *ex vivo* induced a significant MCP-1 protein secretion in wild type mice, the effect was significantly diminished in TLR2_ko_ and TLR4_def_ tissue and blocked in TLR2_ko_/TLR4_def_ aortic tissue (Fig. 4D). The TLR mRNA expression in the mice was proven via PCR (Suppl. Fig. 4).

### NFκB signaling and TLR mediated MCP-1 production

**Figure 5:**
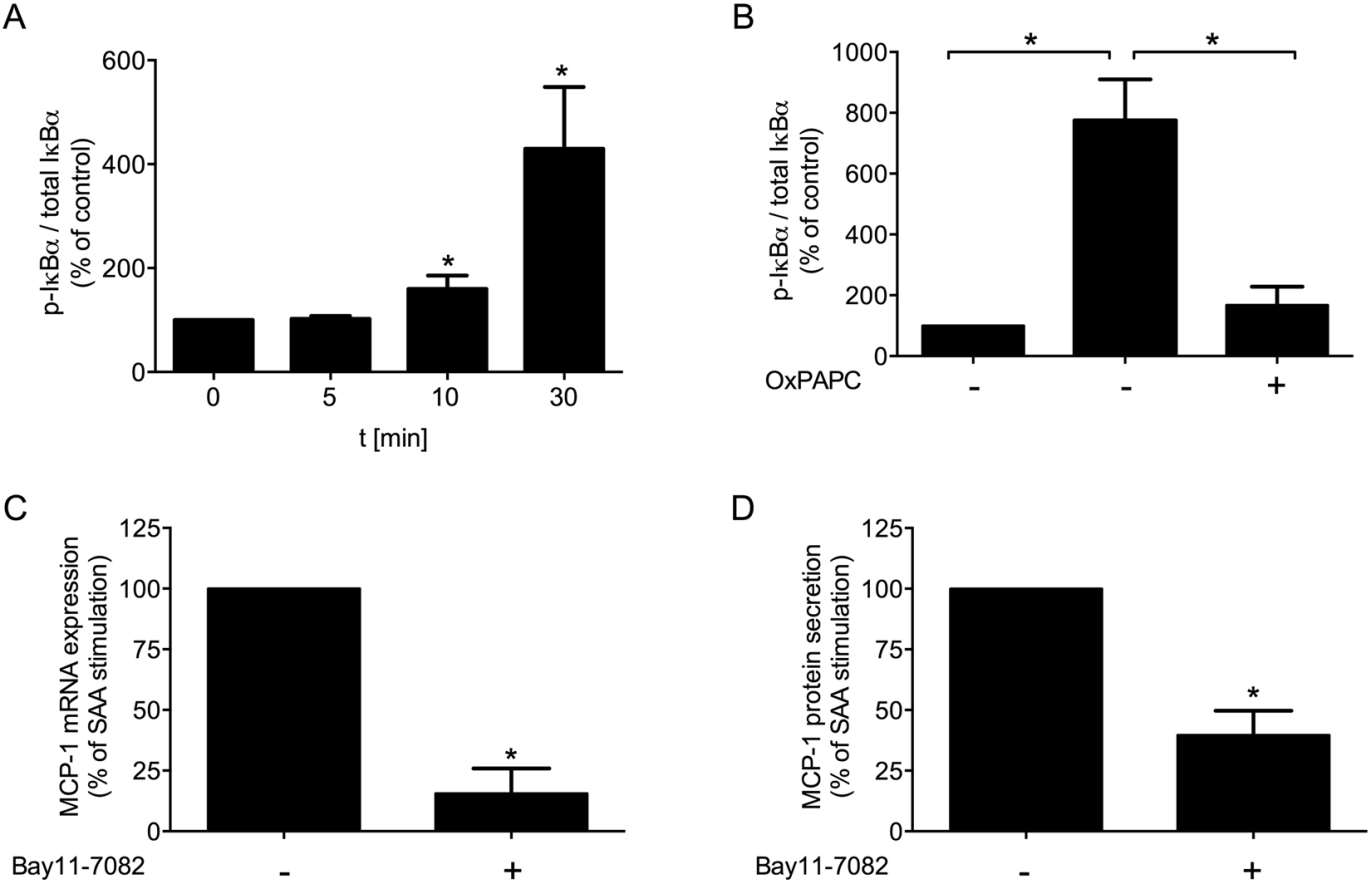
Signalling pathway. (**A**,**B**) Ratio of p-IκBα to total IκBα upon rhSAA (1 µg/ml) stimulation for (**A**) 5, 10, 30 min and (**B**) 30 min in combination with OxPAPC (30 µg/ml). (**C**,**D**) MCP-1 mRNA expression and protein secretion upon rhSAA (1 µg/ml) in co-stimulation with NFκB inhibitor Bay11-7082 (5 µmol/l). (**C**) MCP-1 mRNA expression upon 4 h of stimulation. (**D**) MCP-1 protein secretion upon 24 h of stimulation. Data are presented as means ± SEM. *p < 0.05.

Since NFκB is the intracellular signaling pathway after TLR2 and TLR4 activation, this effect was investigated in the next step. SAA time-dependently stimulated phosphorylation of IκBα with subsequent degradation and translocation of NFkB into the nucleus (Fig. 5A). TLR inhibition with OxPAPC significantly diminished IκBα phosphorylation (Fig. 5B). To confirm that the SAA-mediated MCP-1 production is TLR dependent in rVSMC, cells were pre-incubated with the NFκB inhibitor Bay11–7082 upon stimulation with SAA. MCP-1 mRNA expression and protein secretion was significantly reduced by Bay11-7082 (Fig. 5C,D). These data demonstrate the NFkB-mediated TLR2 and TLR4 activation upon SAA stimulation of the cells.

### HDL-bound SAA induce MCP-1 production in a TLR2- and TLR4-dependent manner

**Figure 6:**
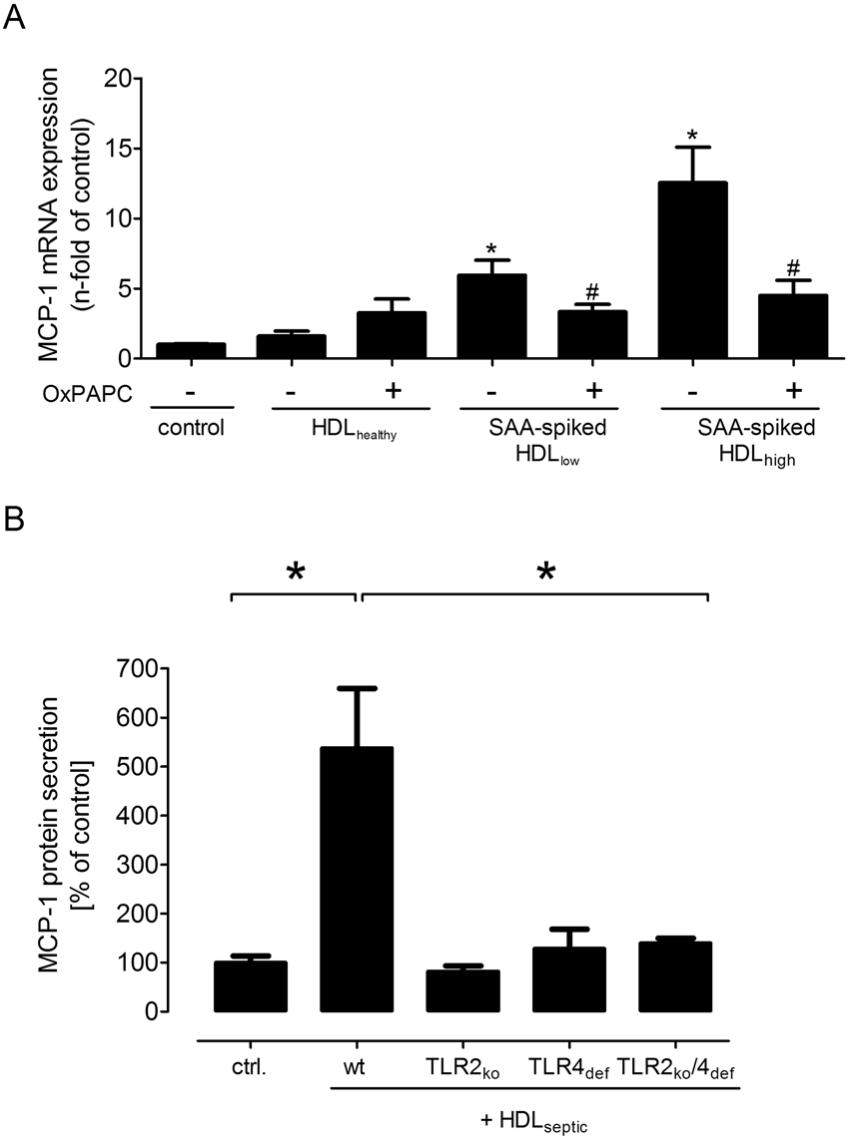
SAA-enriched HDL induced MCP-1 mRNA expression is TLR2 and TLR4 dependent. (**A**) MCP-1 mRNA expression in rVSMC upon HDL (100 µg/ml) and OxPAPC (30 µg/ml) stimulation for 4 h. (**B**) Aortic tissue of TLR2_ko_, TLR4_def_ and TLR2_ko_/TLR4_def_ mice in comparison to control wild type littermates (wt) were stimulated *ex vivo* with HDL (100 µg/ml) isolated from septic patients (HDL_septic_) for 24 h. MCP-1 protein secretion was determined in the supernatant and normalized to the aortic dry weight. Data are presented as means ± SEM. *p < 0.05 compared to control (ctrl.).

Since HDL and SAA-spiked HDL induced MCP-1 production in VSMCs, next, the TLR2 and TLR4-dependency was investigated. As shown in Fig. 6, OxPAPC significantly diminished the MCP-1 mRNA expression for SAA-spiked HDL, whereas no significant influence of the inhibitor was found for HDL from healthy subjects. These data underline the TLR2 and TLR4-mediated signaling pathway for SAA in VSMC. To confer these results for HDL bound SAA, aortic tissue from TLR2_ko_, TLR4_def_ and TLR2_ko/_TLR4_def_ mice were incubated with HDL_septic_ containing a high SAA amount. In accordance to the previous results, *ex vivo* stimulation of aortic rings induced a significant MCP-1 protein secretion in wild type mice with a significant diminished one in TLR2_ko_, TLR4_def_ tissue and TLR2_ko_/TLR4_def_ mice (Fig. 6B).

## Discussion

The present study was designed to investigate the signaling pathway of SAA in vascular cells. The results of this study demonstrate that unbound rhSAA and HDL-bound SAA activates pro-inflammatory acting TLR2 and TLR4 receptors in vascular cells and therefore, SAA exerts direct action on inflammatory conditions.

Several evidence exists for different SAA functions in the acute and chronic inflammatory disorders; however, its precise physiological and pathophysiological role and the associated signaling pathways are not entirely understood. As *in vitro* studies revealed, SAA activates several structurally diverse cell surface receptors namely: FPR2^6,15,16^, TLR2 and TLR4^17–20^, SR-BI^21^ and CD36^22,23^, RAGE^20,24,25^, and P2X_7_^26^. As shown here, all of them are expressed in the rVSMC serving as experimental model in the present study. In a previous study, we have shown the influence of FPR2 for a part of the SAA-induced MCP-1 expression and secretion in rVSMC in a comparable experimental setting^6^. These data are in line with others who found a FPR2-mediated cytokine production upon receptor activation by SAA in vascular cells. Human monocytes and endothelial cells release MCP-1 upon SAA-mediated FPR2 activation leading to subsequent phagocyte migration^15,29,30^. In addition, the chemotaxis of monocytes and mast cells is regulated via FPR2 signaling pathway upon SAA^31,32^. IL-10 and TNFα secretion from monocytes^33^ as well as TNFα and IL-8 secretion from neutrophils^16^ were reported for SAA-mediated FPR2 activation. The current study investigates the meaning of further receptors activated by SAA for the remaining part of the MCP-1 production in rVSMC. Here, data from agonist/antagonist studies, siRNA approach and experiments on aortic tissue from receptor knock-out and deficient mice revealed the influence of TLR2 and TLR4 activation. These data are in line with the literature. SAA-induced cytokine production via activation of TLR2 has been shown in mouse^17,19,26^ and human macrophages^26,34,35^. In a mouse lung model, SAA induced a robust TLR2-mediated neutrophilic inflammation^36^. Comparable results were found in literature for SAA-mediated TLR4 activation in inflammatory pathways. SAA-mediated TLR4 activation leads to a subsequent activation of the inflammasome in human macrophages^26,35^ and induces nitric oxide production in murine peritoneal macrophages^18^. In addition, HMGB1 is released via SAA-mediated TLR4 receptor activation and SAA-neutralizing antibodies are protective against endotoxemia in septic patients^20^.

An influence of the scavenger receptors SR-BI and CD36 could not be found for SAA-induced chemokine production in the investigated experimental setting; however, it could also not be excluded. Unfortunately, for CD36 no selective agonist and antagonist exist so that the current study only investigated the influence of SAA on CD36 mRNA expression, where no effect was found. Baranova and colleagues demonstrated that SAA binds and activates CD36 in a setting with CD36 deficient mouse macrophages^22^. HEK293 cells secreted IL-6 and IL-8 upon SAA via CD36 activation^22^. For SR-BI it has been shown that both lipid-free and HDL-bound SAA are able to bind and activate SR-BI^21^. Subsequently, the receptor is internalized by hepatocytes leading to an inhibition of HDL uptake^21^. Although SR-BI is expressed in rVSMC, the data revealed no influence of this receptor in SAA-mediated MCP-1 production. Neither the mRNA expression of SR-BI is time-dependently influenced by SAA stimulation of rVSMC nor the selective SR-BI inhibitor FPS-ZM1 was able to reduce SAA-induced MCP-1 production. Similar results were found for RAGE activation. Neither a time-dependent regulation of RAGE mRNA expression upon SAA nor an effect of the RAGE antagonist BLT-1 was found in rVSMC regarding MCP-1 production in the current experimental model. In contrast, SAA induced inflammatory cytokine production as IL-6 and IL-8 expression via RAGE activation in synovial fibroblasts^24^. Furthermore, RAGE activation via SAA is involved in the formation of atherosclerotic lesions within the vascular wall^25^. RAGE knock-out animals were protected against uremia-induced acceleration of SAA increase and atherogenesis^25^. As mentioned already for TLR4, the SAA-induced HMGB1 release depends not only on TLR4, but also in RAGE activation^20^. Therefore, a different regulation of SAA induced cytokine production in different vascular cells exists.

Regarding P2X_7_ activation via SAA, it is already known that activation of this purinoceptor leads to activation of the inflammasome in human and mouse macrophages^26^. In the current study however, only an influence of SAA on P2X_7_ expression could be found. Neither a specific P2X_7_ antagonist nor a specific agonist could verify any role of this receptor subtype for MCP-1 production in rVSMC in the current model.

SAA is known for a long time^37,38^ and its predictive function as biomarker has been shown recently for cardiovascular events in patients with diabetes mellitus type 2^39^ and patients with rheumatoid arthritis^40^. Infusion in mice in an experimental model of atherosclerosis leads to an increase in macrophage-specific reverse cholesterol transport and subsequent reduction of the atherosclerotic burden^41^.

As apolipoprotein, SAA accumulated mainly in HDL^42^. However, during acute-phase response, the level of lipid-free SAA increases in serum and beside hepatic tissue, SAA is also synthesized in peripheral tissues by several cell types^1^. As known from previous studies, high concentration of HDL-bound SAA modifies the functionality of HDL from anti-inflammatory to pro-inflammatory properties^6,7^. The current study shows that effects of HDL from septic patients with high SAA plasma concentrations could be replicated by modifying HDL from healthy donors with rhSAA. The pro-inflammatory effect of this modified HDL could be diminished by the inhibition of the TLR2 and TLR4 receptor. Therefore, the data of this study revealed pro-inflammatory actions of lipoprotein-bound SAA and lipid-free SAA by activation of the TLR2 and TLR4 receptor.

However, others found a diminished chemoattractant property of SAA to human macrophages when it is bound to HDL^43^. Moreover, the TNFα production upon SAA was found to be inhibited by HDL in THP-1 cells^44^. As opposed to those findings, HDL was not able to inhibit binding of SAA to the surface of gram-negative bacteria^45^, where SAA aggregates forms channel forming hexamers^46,47^. As shown by Malle *et al*. few years ago^48,49^, the conformation and oligomeric state of SAA seems identical and the same epitopes are exposed in both forms in case of lipid-free and HDL-bound form^48,49^. Why do we observe these divergent results? On the one hand HDL-bound SAA has diminished actions whereas on the other hand full action of HDL-bound SAA could be observed. This cannot be explained easily, but one has to keep in mind that HDL as lipid particle is full of other functional substances that might interfere with SAA actions. This might explain the observed effects. HDL is also known to act as a detoxifying agent. It is speculative but not impossible that HDL absorbs SAA to diminish negative actions of free SAA, but under uremic or septic conditions HDL cannot function properly because of overwhelming production of SAA^50^.

Although many pro-inflammatory actions of SAA are well described in different experimental settings, some anti-inflammatory actions of SAA has also been found^1^. The anti-inflammatory cytokine IL-10 is induced by SAA^17,33,51^, the oxidative stress of neutrophils is reduced^52^, and the inflammatory response in a pneumonia mice model was inhibited by SAA^53^. In addition, the proliferation of regulatory T cells is induced upon SAA^54^. However, the physiological role of these described anti-inflammatory actions of SAA *in vivo* are not clear and could only be speculated.

We cannot fully exclude the influence of other pro-inflammatory acting HDL associated compounds under septic conditions, but the high HDL-associated SAA concentration plays a major role in the pro-inflammatory property of HDL as shown by the artificially enriched SAA within HDL from healthy controls. This effect is comparable with HDL isolations from septic patients.

In summary, our data provide an additional line of evidence that SAA accumulation in HDL reduces its anti-inflammatory capacity by activating pro-inflammatory signaling pathways. SAA plays a key role in decreased HDL functionality and therefore represents an interesting therapeutic target for influencing the fate of cardiovascular disease. However, a direct pharmacological treatment by receptor antagonists might be hampered by the variety of receptors activated by SAA. But, there is now very good evidence that targeting of TLR2 and TLR4 receptors might be of interest.

## Methods

### Materials

All cell culture media, supplements and PBS Dulbecco’s (w/o Ca^2+^/Mg^2+^ pH 7.4) are purchased from Biochrom AG, Berlin, if it’s not mentioned otherwise. All cell culture media were supplemented with 1% (v/v) penicillin/streptomycin. Dulbecco’s Modified Eagle’s medium (DMEM) and Rosewell Park Memorial Institute (RPMI) media were supplemented with 10% (v/v) heat-inactivated fetal bovine serum. The digestion solution TrypLE™ Express and RPMI media were purchased from Gibco® by Live Technologies, Karlsruhe.

Substances: recombinant human SAA (rhSAA, contains SAA1 and SAA2, Peprotech, Hamburg, Germany), LPS (Sigma-Aldrich, Munich, Germany), HKSA (InvivoGen, Toulouse, France), Bz-ATP (Sigma-Aldrich, Munich, Germany), OxPAPC (InvivoGen, Toulouse, France), KN-62 (Sigma-Aldrich, Munich, Germany), CLI-095 (TAK-242, InvivoGen, Toulouse, France), Bay11-7082 (Calbiochem, MerckMillipore, Darmstadt, Germany).

### Animals

This study was carried out in accordance with the recommendation in the Guide for the Care and Use of Laboratory Animals and was approved by the Landesamt fuer Gesundheit und Soziales Berlin, Germany. Explantation of aortic tissue was performed under sodium pentobarbital anesthesia (400 mg/kg body weight, intraperitoneal). For VSMC isolation, Wistar rats were used. For the *ex vivo* experiments, the following mouse strains were used: C57BL/10ScN, which are naturally deficient for TLR4^55,56^, C57BL/10ScSn-TLR2tm1, which have an induced knockout for TLR2^57,58^, C57BL/10ScSn-TLR4/TLR2, where mice with induced TLR2 knockout were crossed with naturally TLR4 deficient mice, and C57BL/10ScSn as control mice.

### HDL isolation

HDL was isolated of human blood. The blood sampling has been carried out in accordance with the Declaration of Helsinki of the World Medical Association, and has been approved by the local Ethics Committee of the Charité, Berlin, Germany. All participants signed an informed consent for study participation. Blood of healthy control subjects and sepsis patients was used for experiments. HDL was isolated from human serum as described previously^6^. For indicated experiments, HDL of healthy control subjects was spiked with SAA. Therefore, HDL was incubated for 4 h at 37 °C with 25 µg/ml and 100 µg/ml rhSAA. Afterwards, the sample was centrifuged repeatedly using a cutt-off filter to separate unbound SAA from HDL-bound SAA. For the experiments, only the HDL-bound SAA was used. Concentrations of SAA were checked using SAA ELISA (IBL, Germany) according to the manufacturer´s instructions.

### Isolation of rat vascular smooth muscle (cells from rat aortas)

Wistar rats were euthanized by an overdose of pentobarbital. The thoracic aorta was cut from the arch to the diaphragm under aseptic conditions, cleaned, infused by phosphate-buffered saline (PBS) buffer (37 °C) and the adventitial layer was removed using a microscissor. rVSMC were isolated by explant outgrowth method as described previously^59^.

### Cell culture

rVSMC were cultured in DMEM (1.0 g/L glucose) supplemented with 10% fetal bovine serum (FBS), penicillin (100 U/mL), streptomycin (100 µg/mL), and stable L-glutamine. Cells were maintained at 37 °C in a humidified atmosphere with 5% CO2 in air. THP-1 cells were obtained from the DSMZ Braunschweig, Germany (ACC 16). Cells were cultured in RPMI 1640 media containing 10% fetal calf serum, 1% antibiotics. THP-1 monocyte differentiation into macrophages was induced by treatment with phorbol-12-myristate-13-acetate (PMA) for 24 h^60^.

### RNA isolation and reverse transcription PCR analysis

Total RNA from rVSMC was isolated using RNeasy® Mini Kit (Qiagen, Hilden, Germany) following the manufacturer’s instructions. The purity and amount of RNA were determined by measuring the OD at the ratio of 260 to 280 nm via NanoDrop photometer (Thermo Fisher Scientific, Dreieich, Germany). RNA was reverse transcribed into cDNA using the High Capacity cDNA Reverse Transcription Kit (Applied Biosystems/Life Technologies, Darmstadt, Germany) following the manufacturer’s instructions. The purity and amount of cDNA were determined by measuring the OD at the ratio of 260 to 280 nm via NanoDrop photometer.

### Detection of MCP-1 production

For the gene expression studies, rVSMC were serum starved for 24 h prior stimulation. Afterwards, cells were stimulated for 4 h with agonists. In case of antagonistic studies, antagonists were pre-incubated for 30 min. MCP-1 mRNA expression was determined by quantitative real-time PCR using the CFX96/384 System and appropriated SybrGreen kit (Bio-Rad, Muenchen, Germany). The oligonucleotides were synthesized by TibMolBiol (Berlin, Germany). The oligonucleotide sequences are specified in the Supplementary Table 1. Quantification of gene expression was normalized using β-actin as a house keeping gene.

MCP-1 protein secretion was measured in supernatants of stimulated cells (rVSMC, THP-1) and *ex vivo* stimulation of aortic rings. Stimulation was performed for 24 h. Cells were serum-starved for 24 h prior stimulation. After stimulation, supernatants were collected for quantification of MCP-1 protein secretion. MCP-1 protein was measured with the Milliplex® map kit for rat or human MCP-1 protein (Millipore, Schwalbach, Germany) according to manufacturer’s instruction using Luminex™ technique (BioRad, Muenchen, Germany). MCP-1 secreted protein was normalized to the total protein content of the lyzed cells or aortic dry weight.

### Cell transfection for TLR2 and TLR4 silencing

Cells were serum starved for 48 h prior gene silencing experiments. Rat aortic VSMCs were trypsinized, counted, resuspended in transfection solution and were transfected with pre-designed small interference RNA (siRNA) against TLR2 and TLR4 (Applied Biosystems/Life Technologies, Darmstadt, Germany) or non-related scrambled nucleotides with similar GC content (Invitrogen/Life Technologies, Darmstadt, Germany) by electroporation using the Amaxa Nucleofector II system and Amaxa Basic Nucleofector kit solution for primary VSMC (Lonza, Koeln, Germany). The knockdown efficiency of siRNA on TLR2 and TLR4 mRNA expression is ~80% (Suppl. Fig. 5).

### Detection of signaling molecule IkB-a

Cultured cells were serum starved for 48 h prior to stimulation. VSMCs were stimulated for 1 to 30 min with SAA and OxPAPC. The antagonist was pre-incubated for 30 minutes. For the quantification of phosphorylated proteins, cells were washed with ice-cold PBS and lyzed using a buffer included in the kit (Bio-Plex® Cell Lysis Kit). Total content of IkB-a and phosphorylated IkB-a (p-IkB-a) was measured with the Bio-Plex® Phosphoprotein Detection Kit (BioRad, Muenchen, Germany) according to manufacturer´s instruction using Luminex™ technique (BioRad, Muenchen, Germany). The phosphorylated protein was normalized to the total protein content of the lyzed cells.

### Statistical analysis

Data are provided as mean ± SEM. The experiments were repeated at least in 3 independent experiments or in the number as indicated. Statistical analysis was performed using GraphPad Prism software (version 5.0). To evaluate differences between treatment groups Mann-Whitney U test or Wilcoxon matched paired test were applied. A p value < 0.05 was considered as statistically significant.

## Supplementary information

Supplementary Information
