# Supplementary material for: Dysfunctional high-density lipoprotein activates toll-like receptors via serum amyloid A in vascular smooth muscle cells
Source: Sci Rep. 2019 Mar 4;9:3421. doi: 10.1038/s41598-019-39846-3 (PMC6399289; doi:10.1038/s41598-019-39846-3)
Supplement: Supplementary file 1 — Supplementary Information [file 41598_2019_39846_MOESM1_ESM.docx]

**Dysfunctional high-density lipoprotein activates toll-like receptors via serum amyloid A in vascular smooth muscle cells**

Mirjam Schuchardt^1,*^, Nicole Prüfer^1,*^, Yuexing Tu^1,2^, Jaqueline Herrmann^1^, Xiu-Ping Hu^1^, Sarah Chebli^1^, Katja Dahlke^3^, Walter Zidek^1^, Markus van der Giet^1,#^, Markus Tölle^1^

*authors contributed equally

**SUPPLEMENTARY MATERIAL**

**METHODS**

**Analysis of receptor expression**

The existence of the analyzed receptors in rVSMCs was determined by semi-quantitative PCR and subsequent agarose gel electrophoresis.

The following protocol was used for detection of receptor expression in rVSMC. Here, the ready-to-use solution (containing bacterially derived Taq DNA polymerase, dNTPs, MgCl_2_ and reaction buffers) GoTaq® Green Master Mix 2x (Promega, Mannheim, Germany) was used. Template, primers and nuclease-free water were added to a total reaction volume of 25 µl. The PCR program includes following steps: 2 min 95° C initial denaturation, 45 sec 95° C denaturation, 45 sec 60° C annealing, 60 sec 72° C extension (35 cycles) and 5 min 72° C final extension. The oligonucleotides were synthesized by TibMolBiol (Berlin, Germany). The sequences are specified in Table 1.

For detection of TLR2 and TLR4 in mice, the following mice with different genotypes were used: C57BL/10ScSn-TLR2^tm1^, C57BL10ScN-TLR4/TLR2^tm1^, C57BL/10ScN-TLR4 (natural TLR4 deficient ^1^) and C57BL/10ScSn (wild type). Breeding pairs of these mice were kindly provided by Dr. Marina Freudenberg (2003, Max-Plank Institute, Freiburg, Germany) and were maintained as a breeding colony under specified pathogen-free conditions in the animal facility (MRL) of the German Institute of Human Nutrition.

The presence (TLR2 deficiency by insert of neomycin-resistant gene) and absence of mRNA (TLR4 natural lack) for TLR2 and TLR4 in C57BL/10ScSn-TLR2^tm1^, C57BL/10ScN-TLR4, C57BL10ScN-TLR4/TLR2^tm1^ and C57BL/10ScSn (wild type) mice was verified by semi-quantitative PCR with subsequent agarose gel electrophoresis. To confirm the TLR expression/deficiency, total RNA was extracted from the kidney tissue using peqGold TriFast^TM^ reagent (PEQLAB Biotechnologie GmbH, Erlangen, Germany), according to the manufacturer's instructions. Optical density (OD) was measured using ND-1000 spectrophotometer (PEQLAB Biotechnologie GmbH, Erlangen, Germany) to determine RNA concentrations and purity. 5 µg of total RNA from each sample were converted to cDNA using RevertAid RT Reverse Transcription Kit^TM^ (Thermo Fischer Scientific, Germany) according to the manufacturer's instructions using Oligo(dT)_18_ Primer. The TLR2 allele was PCR amplified using either a common fwd primer with a wild type rev primer specific for the TLR2 gene or the common fwd primer in combination with a specific rev primer for the neomycin-resistant gene used for the targeting construct (specified in Tab. 1). PCR was performed in a total volume of 25 µl in PCR buffer in the presence of 0.2 mmol/l dNTP, 20 µM of each primer, 2 mmol/l MgCl_2_, and 2.5 units HotStarTaq *Plus* DNA Polymerase (Qiagen, Valencia, CA, USA). PCR amplification was conducted by an initial hot start at 95° C for 5 min followed by 10 cycles of 25 sec 95°C denaturation, annealing temperature stepdown every cycle of 0.5°C (from 65°C); 40 sec 68°C extension followed by 28 cycles 20 sec 95°C denaturation, 30 sec 60°C annealing, 40 sec 72°C extension and 5 min 72°C final extension. To show the natural deletion of the tlr-4 gene that results in the absence of mRNA in C57BL/10ScN-TLR4 and C57BL10ScN-TLR4/TLR2^tm1^ mice a conventional PCR was performed in a total volume of 25 µl in presence of PCR buffer, 0.2 mmol/l dNTP, 20 µmol/l of each primer (specified in Tab. 1), 2 mmol/l MgCl_2_, and 2.5 units HotStarTaq *Plus* DNA Polymerase (Qiagen, Valencia, CA, USA). PCR conditions for this primer couple were as follows: 5 min 95°C initial denaturation, 45 sec 95°C denaturation, 45 sec 64°C annealing, 45 sec 72°C extension (34 cycles) and 10 min 72°C final extension. The oligonucleotides were synthesized by Eurofins Genomics (Ebersberg, Germany).

**Cell viability assay**

The viability of VSMCs was determined by MTS CellTiter 96® AQueous One Solution Cell Proliferation Assay from Promega (Mannheim, Germany). Cells were seeded in 96-well plates, serum-starved for 24 h and afterwards stimulated with agonists and antagonists for 4 and 24 h in serum-free medium. Ionomycin as the positive control was added simultaneously with substrate 30 min prior measurement. Absorbance was measured at 492 nm.

**RESULTS**

**Receptor expression in rVSMC**

At least seven receptors have been identified to be activated by SAA: formyl-peptide receptor-like 1 (FPR2), toll-like receptor 2 and 4 (TLR2/4), scavenger receptor type B-I (SR-BI) and CD36, receptor for advanced glycation end-products (RAGE), and the purinoceptor P2X_7_ ^2^. All of these receptors were expressed in rVSMC (suppl. Figure 1). In contrast to the mRNA expression increase of P2X_7_, TLR2 and TLR4 upon SAA stimulation (Figure 2), the mRNA expression of CD36, RAGE and SR-BI is not influenced by SAA stimulation of rVSMC (suppl. Figure 2 A, B, C). In addition, neither inhibition of RAGE using FPS-ZM1 (0.5 µmol/l) nor inhibition of SR-BI using BLT-1 (100 nmol/l) reduced SAA-induced MCP-1 mRNA expression in rVSMC (suppl. Figure 2 D, E).

**Viability of rVSMC following exposure to the agonists and antagonists**

In order to evaluate any potential viability effects of the agonists and antagonists, cell viability assay based on metabolism was performed and showed no significant changes. VSMC were stimulated with the agonists: SAA (1 µg/ml), BzATP (100 µmol/l), LPS (1 µg/ml), HKSA (10 µg/ml) and S100B (10 µg/ml) (suppl. Fig. 3 A, C) and antagonists: Bay 11-7082 (5 µmol/l), WRW4 (10 µmol/l), OxPAPC (30 µg/ml), CLI-095 (1 µg/ml), BLT-1 (100 nmol/l) and KN-62 (10 µmol/l) (suppl. Fig. 3B and 3D). Cell viability was measured 4 h and 24 h after treatment, respectively. As expected, ionomycin (10 mmol/l) as positive control impaired the cell viability. Similar results were found for THP-1 cells stimulated with SAA and LPS for 24 h and oxPAPC and CLI-095, respectively (suppl. Figure 3 E and 3 F).

**Receptor expression of TLR2 and TLR4 in mice**

All mice strains used were screened for TLR2 and TLR4 mRNA via cDNA synthesis with subsequent PCR. Mice deficient in TLR2 generated by gene targeting with an insert of neomycin-resistant gene that disrupts the TLR2 gene, as described by Werts *et al.* ^3^ (C57BL/10ScSn-TLR2^tm1^) and TLR2/TLR4 double-deficient mice (C57BL10ScN-TLR4/TLR2^tm1^) revealed only the TLR2 gene with neomycin-resistant insert whereas only wild type PCR products are detectable in the other strains (Suppl. Fig. 4).

Compared to animals with wildtype TLR4 expression (C57BL/10ScSn-TLR2^tm1^; C57BL/10ScSn) C57BL10ScN-TLR4/TLR2^tm1^ and C57BL/10ScN-TLR4 mice carrying a natural homozygous TLR4 defect (TLR4^−/−^), showed no yield of any PCR product (Suppl. Fig. 4).

**Silencing of TLR2 and TLR4 receptor in rVSMCs using siRNA**

Under basal conditions, TLR2 mRNA expression is low in rVSMC used in this study. Under SAA stimulation (1 µg/mL), both the TLR2 and the TLR4 receptor expression are stimulated in a time-dependent manner (Figure 2). Under stimulated condition with SAA or the respective receptor agonist (10^7^ cells/mL HKSA, 1 µg/mL LPS), the TLR2 as well as the TLR4 mRNA expression could be significantly silenced by siRNA with an efficiency of ≈ 80% (Suppl. Fig 5).

**REFERENCES**

1. Vogel, S. N., Hansen, C. T. & Rosenstreich, D. L. Characterization of a congenitally LPS-resistant, athymic mouse strain. *J Immunol* **122**, 619-622 (1979).

2. Ye, R. D. & Sun, L. Emerging functions of serum amyloid A in inflammation. *J Leukoc Biol* **98**, 923-929 (2015).

3. Werts, C. *et al.* Leptospiral lipopolysaccharide activates cells through a TLR2-dependent mechanism. *Nat Immunol* **2**, 346-352 (2001).

4. Schuchardt, M. *et al.* The endothelium-derived contracting factor uridine adenosine tetraphosphate induces P2Y(2)-mediated pro-inflammatory signaling by monocyte chemoattractant protein-1 formation. *J Mol Med (Berl)* **89**, 799-810 (2011).

5. Liu, N., Liu, J., Ji, Y. & Lu, P. Toll-like receptor 4 signaling mediates inflammatory activation induced by C-reactive protein in vascular smooth muscle cells. *Cell Physiol Biochem* **25**, 467-476 (2010).

6. Syed, N. I., Tengah, A., Paul, A. & Kennedy, C. Characterisation of P2X receptors expressed in rat pulmonary arteries. *Eur J Pharmacol* **649**, 342-348 (2010).

7. Chen, C. Y., Abell, A. M., Moon, Y. S. & Kim, K. H. An advanced glycation end product (AGE)-receptor for AGEs (RAGE) axis restores adipogenic potential of senescent preadipocytes through modulation of p53 protein function. *J Biol Chem* **287**, 44498-44507 (2012).

8. Laboratory, T. J. *Protocol Search 004650*, <https://www2.jax.org/protocolsdb/f?p=116:5:0::NO:5:P5_MASTER_PROTOCOL_ID,P5_JRS_CODE:14512,004650> (2015).

9. Wolfs, T. G. *et al.* In vivo expression of Toll-like receptor 2 and 4 by renal epithelial cells: IFN-gamma and TNF-alpha mediated up-regulation during inflammation. *J Immunol* **168**, 1286-1293 (2002).

**TABLE**

Table S1: Oligonucleotide sequences used for semiquantitative- and Real-time PCR

| Species | Target Gene | Oligonucleotide sequence (5´ - 3´) | Amplicon [bp] | reference |
| --- | --- | --- | --- | --- |
| rat | *β-actin* | Fwd: TCG CTG ACA GGA TGC AGA AG  Rev: CTC AGG AGG AGC AAT GAT CTT GAT | 76 | ^4^ |
| rat | *mcp-1* | Fwd: CTG TCT CAG CCA GAT GCA GTT AAT  Rev: TTC TCC AGC CGA CTC ATT GG | 86 | ^4^ |
| rat | *tlr-2* | Fwd: GGT CTC CAG GTC AAA TCT CAG AGG A  Rev: CGG AGG TTC ACA CAG GCT CGC | 260 | NM_198769.2 |
| rat | *tlr-4* | Fwd: GGC ATC ATC TTC ATT GTC CTT G  Rev: AGC ATT GTC CTC CCA CTC G | 111 | ^5^ |
| rat | *cd36* | Fwd: TGC ATG AAT TAG TTG AAC CAG GCC A  Rev: CGG AGA GGT ACC AGA TGG GA | 127 | NM_031561.2 |
| rat | *sr-b1* | Fwd: CTC TCA GCG GAC AAC CCG CC  Rev: TGC TTG TCC AGC CGT GAC GC | 333 | NM_031541.1 |
| rat | *p2x7* | Fwd: AAT GAG TCC CTG TTC CCT GGC TAC  Rev: CAG TTC CAA GAA GTC CGT CTG G | 468 | ^6^ |
| rat | *fpr2* | Fwd: GGC TGG TTT CTG TGT AAA TTA G  Rev: TCA GAG CTA AAA TCC AGG G | 184 | NC_005100.2 |
| rat | *rage* | Fwd: CAG GGT CAC AGA AAC CGG  Ref: ATT CAG CTC TGC ACG TTC CT | 214 | ^7^ |
| murine | *tlr-2*  *neo* | Common: CTT CCT GAA TTT GTC CAG TAC A  WT: ACG AGC AAG ATC AAC AGG AGA  Mutant: GGG CCA GCT CAT TCC TCC CAC | WT: 499 Mutant: 334 | ^8^ |
| murine | *tlr-4* | Fwd: GCA ATG TCT CTG GCA GGT GTA  Rev: CAA GGG ATA AGA ACG CTG AGA | 406 | ^9^ |
| murine | *β-actin* | Fwd: TAA AAC GCA GCT CAG TAA CAG TCG G  Rev: TGC AAT CCT GTG GCA TCC ATG AAA C | 320 | ^9^ |

fwd: forward primer, rev: reverse primer, WT: wild type

**FIGURE LEGENDS**

**Suppl. Figure 1 mRNA expression of receptors activated by SAA.**

RNA from rVSMC was isolated, reverse transcribed and amplificated with specific oligonucleotides for the different receptors. PCR products were separated via ethidiumbromid-stained agarose gel electrophoresis.

**Suppl. Figure 2 CD36, RAGE and SR-BI upon SAA-incubation.**

rVSMC were stimulated with SAA (1 µg/ml) for 4, 24 and 48 h. mRNA expression of (A) CD36, (B) RAGE, and (C) SR-BI upon SAA stimulation. (D) Effect of RAGE antagonist FPS-ZM1 (0.5 µmol/l) on SAA-induced MCP-1 mRNA expression. (E) Effect of SR-BI antagonist BLT-1 (100 nmol/l) on SAA-induced MCP-1 mRNA expression. Data are presented as mean ± SEM.

**Suppl. Figure 3 Cell viability upon treatment with agonists and antagonists.**

(A-D) rVSMC and (E-H) THP-1 were stimulated with agonists: SAA (1 µg/ml), BzATP (100 µmol/l), LPS (1 µg/ml), HKSA (10 µg/ml) and S100B (10 µg/ml) and antagonists: Bay 11-7082 (5 µmol/l), OxPAPC (30 µg/ml), CLI-095 (1 µg/ml), BLT-1 (100 nmol/l) and KN-62 (10 µmol/l) for 4 or 24 h in serum free medium. Cell viability was measured by the amount of MTT reduction and OD value at 490 nm. Results are shown as % of unstimulated control cells (crtl). Data are presented as mean ± SEM.

**Suppl. Figure 4 TLR mRNA expression in mice.**

TLR2 and TLR4 mRNA expression was evaluated by PCR amplification of renal cDNA samples of mice (TLR2^-/-^: C57BL/10ScSn-TLR2^tm1^; TLR2+4^-/-^:C57BL10ScN-TLR4/TLR2^tm1^; TLR4^-/-^: C57BL/10ScN-TLR4; and wild type: C57BL/10ScSn) and no template control (NTC). Complementary DNA from mice with TLR2^-/-^ yielded the expected 334 bp product whereas the other strains revealed the expected wild type product with 499 bp. C57BL10ScN-TLR4/TLR2^tm1^ and C57BL/10ScN-TLR4 (lane 8 and 9) show that these strains produces no detectable TLR4 (natural lack) mRNA in contrast to the corresponding strains with TLR4 amplification products of 406 bp.

**Suppl. Figure 5 Efficiency of siRNA on TLR2 and TLR4 mRNA expression.**

rVSMC were transfected with (A) TLR2 and (B) TLR4 selective siRNA under stimulation with SAA (1 µg/mL), HKSA (10^7^ cells/mL), or LPS (1 µg/mL). Results are shown as % of control (ctrl.). Data are presented as mean ± SEM. *p<0.05
